# Genetic analysis of morphological traits in spring wheat from the Northeast of China by a genome-wide association study

**DOI:** 10.3389/fgene.2022.934757

**Published:** 2022-08-18

**Authors:** Wenlin Liu, Yuyao Li, Yan Sun, Jingquan Tang, Jingyu Che, Shuping Yang, Xiangyu Wang, Rui Zhang, Hongji Zhang

**Affiliations:** ^1^ Crop Resources Institute, Heilongjiang Academy of Agricultural Sciences, Harbin, China; ^2^ Heilongjiang Academy of Agricultural Sciences, Harbin, China; ^3^ KeShan Branch of Heilongjiang Academy of Agricultural Sciences, Qiqihaer, China; ^4^ Institute of Forage and Grassland Sciences, Heilongjiang Academy of Agricultural Sciences, Harbin, China

**Keywords:** GWAS, marker-assisted selection, *Triticum aestivum* L, spring wheat, morphological traits

## Abstract

Identification of the gene for agronomic traits is important for the wheat marker-assisted selection (MAS) breeding. To identify the new and stable loci for agronomic traits, including flag leaf length (FLL), flag leaf width (FLW), uppermost internode length (UIL), and plant morphology (PM, including prostrate, semi-prostrate, and erect). A total of 251 spring wheat accessions collected from the Northeast of China were used to conduct genome-wide association study (GWAS) by 55K SNP arrays. A total of 30 loci for morphological traits were detected, and each explained 4.8–17.9% of the phenotypic variations. Of these, 13 loci have been reported by previous studies, and the other 17 are novel. We have identified seven genes involved in the signal transduction, cell-cycle progression, and plant development pathway as candidate genes. This study provides new insights into the genetic basis of morphological traits. The associated SNPs and accessions with more of favorable alleles identified in this study could be used to promote the wheat breeding progresses.

## Background

Common wheat (*Triticum aestivum* L.) is an essential cereal crop and provides nearly 20% of the total caloric input to the global population (He et al., 2010; Wang et al., 2019). Due to the complicated genetic architecture (Gao et al., 2017; Li et al., 2021), the progresses for the improvement of morphological traits are difficult. Heilongjiang and Jilin provinces located at the Northeast of China are the major spring wheat-producing regions (Li et al., 2021). Although the wheat production has been improved largely in the past decades, it is still not enough to meet the needs of the people (He et al., 2010; Xu et al., 2021).

Morphological traits include various traits, such as flag leaf length (FLL), flag leaf with (FLW), uppermost internode length (UIL), and plant morphology (PM, including prostrate, semi-prostrate, and erect) (Li et al., 2018; Li et al., 2021). Both the genetic and environmental factors influenced morphological traits. Marker-assisted selection (MAS) based on molecular marker is an available and effective approach for the further improvement of morphological traits of wheat (He et al., 2010; Rasheed et al., 2016; Wang et al., 2020). However, the effective and reliability of MAS depend on the number and quality of available genes/QTLs and associated markers for target traits. Until now, over 30 genes associated with morphological traits were cloned by homologous cloning or map-based cloning, and over 50 functional markers or kompetitive allele-specific PCR (KASP) markers were developed (Cui et al., 2014; Rasheed et al., 2016; Nadolska-Orczyk et al., 2017; Lozada et al., 2019). Besides, over 120 loci for morphological traits were detected by linkage mapping and association analysis (Gao et al., 2017; Würschum et al., 2017; Rahimi et al., 2019; Pang et al., 2020; Li et al., 2021). However, the loci/gene is still not enough for the improvement of wheat morphological traits. Identifying the novel genes or loci for morphological traits is urgent.

Single nucleotide polymorphism (SNP), insertion and deletion (InDel), simple sequence repeats (SSRs), and diversity array technology (DArT) markers are the mostly used molecular marker for genotyping (Wang et al., 2014; Rasheed et al., 2016). Compared with SSR and DArT markers, SNPs have more abundant and higher coverage. With the development of the gene chip and NGS technology, getting SNP quickly becomes feasible and provides an effective way to identifying genes/QTLs for complex traits (Zhu et al., 2008; Liu et al., 2017; Wang et al., 2020). Now, the 55, 90, and 660K wheat SNP arrays are gradually replacing SSR and DArT markers in genetic analysis and have been widely used in the genetic analysis for yield (Gao et al., 2015; Sun et al., 2017; Beyer et al., 2019; Li et al., 2019), disease resistance (stripe rust, leaf rust, or powdery mildew), end-use quality, procession quality, and biotic or abiotic stress tolerance (drought or flood)-related traits (Liu et al., 2016a; Liu et al., 2017; Valluru et al., 2017; Liu et al., 2019; Li et al., 2021; Quan et al., 2021).

Linkage analysis and association mapping are the two main ways to uncover the genetic mechanism of complex traits (Zhu et al., 2008; Liu et al., 2016b; Liu et al., 2017). Compared with the bi-parental linkage analysis, association mapping is based on natural population (including wild types, landraces, released cultivars, and improved accessions) and offers an effective and reliable approach to uncover the genetic architecture of complex traits (Liu et al., 2017; Wang et al., 2020). Besides, linkage analysis focused on specific traits, whereas the GWAS could be used to analyze all traits based on the same set of genotype data (Zhu et al., 2008). Nowadays, association mapping has been widely used in the genetic analysis of complex traits in wheat, including grain yield-related traits, disease-related traits, and biotic and abiotic stresses (Cui et al., 2014; Zuo et al., 2020; Quan et al., 2021). The rapid, efficient, and accurate genotyping is the basis to conduct GWAS. Thus, the SNP chip provides an effective and feasible way for association mapping.

In this study, 251 spring wheat accessions mainly collected from the Northeast of China (Heilongjiang and Jilin province) were used to 1) identify loci for morphological traits in spring wheat, 2) get new insights into the genetic architecture of target traits, and 3) search for candidate genes for further study.

## Materials and methods

### Plant materials and field trials

A total of 251 spring wheat accessions from the Northeast of China (Heilongjiang or Jilin province) were collected for the GWAS of morphological traits (Supplementary Table S1). The diverse panel was grown at the Harbin and Keshan experimental station of the Heilongjiang academy of agricultural science in Heilongjiang province during the 2018–2019 and 2019–2020 cropping seasons. A complete randomized block design with three replicates was employed in field trials. For both Harbin and Keshan experimental stations, each plot comprised four 2.0 m rows spaced 20 cm apart, with 40 seeds in each row. Agronomic management was performed according to local practices.

### Phenotyping and statistical analysis for flag leaf length, flag leaf width, uppermost internode length, and plant morphology

Four traits related to morphological were conducted in all four environments, including FLL, FLW, UIL, and PM. For FLL and FLW, 10 random flag leaves in each plot at the mid-grain-fill stage were used to measure FLL and FLW, represented by the distance between the base and the tip, and width at the widest point, respectively. UIL is the mean distance between the stem base and the top of spikes excluding awns and the mean length of the uppermost internode. Ten single plants in each plot were randomly selected at physiological maturity for measuring UIL. BLUE for four traits among four environments was calculated by a one-stage approach using the R package sommer (https://cran.r-project.org/web/packages/sommer/index.html) as $y=1_{n}\mu +\mathrm{Xg}+\mathrm{Ze}+v+\varepsilon$ , where y is the n-dimensional multi-environment phenotypic records, n is the number of multi-environment phenotypic records, $1_{n}$ is an n-dimensional vector of ones, μ is the intercept, g and e are the vector of genetic and environment effects, respectively, v is the vector of genotype-by-environment interaction effects, X and Z are the design matrices of g and e , respectively, and ε is the random residuals. g , e , v , and ε were all assumed as random effects following normal distributions.

### Genotyping, population structure, and linkage disequilibrium

A total of 3000 polymorphic and evenly distributed markers on 21 chromosomes were used to conduct population structure analysis by Structure v2.3.4 (Pritchard et al., 2000) (http://pritchardlab.stanford.edu/structure.html). Besides, principal component analysis (PCA) and neighbor-jointing (NJ) trees were also estimated using the Tassel v5.0 to validate the results of population structure analysis (Breseghello and Sorrells, 2006). The LD decay analysis was calculated for the whole genomes using the full matrix and sliding window options in Tassel v5.0 (Breseghello and Sorrells, 2006). The results of LD decay, population analysis, PCA, and NJ-tree analysis for the 251 spring wheat accessions have been reported by Li et al. (2021). The multi-environment phenotypic data were analyzed by a one-stage approach as $y=1_{n}\mu +\mathrm{Xg}+\mathrm{Ze}+v+\varepsilon$ , where y is the n-dimensional multi-environment phenotypic records, n is the number of multi-environment phenotypic records, $1_{n}$ is an n-dimensional vector of ones, μ is the intercept, g and e are the vector of genetic and environment effects, respectively, v is the vector of genotype-by-environment interaction effects, X and Z are the design matrices of g and e , respectively, and ε is the random residuals. g , e , v , and ε were all assumed as random effects following normal distributions. The heritability was estimated using the entry-mean basis formula $h^{2}=\frac{\sigma_{g}^{2}}{\sigma_{g}^{2}+\frac{\sigma_{v}^{2}}{n_{e}}+\frac{\sigma_{\varepsilon }^{2}}{n_{e}\times n_{r}}}$ , where $\sigma_{g}^{2}$ , $\sigma_{v}^{2}$ , and $\sigma_{\varepsilon }^{2}$ are the variance components of genetic effect, environment effect, and residual, $n_{e}$ and $n_{e}$ are the number of environments and number of replicates per accession in each environment, respectively. The one-stage phenotypic analysis was realized using the R package sommer (https://cran.r-project.org/web/packages/sommer/index.html) in statistical software R. We have added the statistical method in the M&M section. The SNP-based heritability was estimated by the GREML-LDMS method based on GCTA (Yang et al., 2011) (https://yanglab.westlake.edu.cn/software/gcta/) software.

### Genome-wide association study and the identification of candidate genes

To eliminate the spurious marker-trait associations (MTAs) caused by environment variation, the mixed linear model (MLM, PCA + K) in Tassel v5.0 (Breseghello and Sorrells, 2006) was used as follows: *y* = *μ* + *xβ* + *u* + *e*. Of these, *y* is the vector of phenotype; *µ* is the mean; *x* represents the genotype; *β* is the effect of the SNP; and *u* is the random effects. The kinship matrix was treated as a random-effect factor, whereas the PCA was considered as a fixed-effect factor. Both the kinship and PCA matrix were calculated by the software Tassel v5.0. In this study, markers with a -log_10_ (*p*-value) ≥ 3.0 were regarded as MTAs. Manhattan plots and Q–Q plots were drawn by the CMplot package (https://cran.r-project.org/web/packages/CMplot/index.html) based on R 3.6.5.

The flanking sequences corresponding to the SNP markers (the SNPs in the LD decay interval) significantly associated with morphological traits were used in BLASTn and BLASTx searches against NCBI (http://www.ncbi.nlm.nih.gov/) databases. Besides, the annotation for IWGSC v2.1 was also used to identify candidate genes. Potential candidate genes were then selected with significant MTAs corresponding to non-synonymous SNPs in the coding region of the genes.

Quantitative real-time PCR (qRT-PCR) was conducted to test expression differences of the candidate genes in the accessions with extreme traits (Supplementary Table S2). The flag leaf used for test FLL and FLW was sampled for RNA extraction during the flag leaf fully drawn out; the stem used for test PM was sampled for RNA extraction during the erecting period; the uppermost internode used for test UIL was sampled for RNA extraction during the heading stage. Total RNA was extracted according to the Trizol method, whereas cDNA was synthesized with the HiScript II 1st Strand cDNA Synthesis Kit (Vazyme, Nanjing, China). The primers were designed with Primer Premier 5.0 software9 (Supplementary Table S3). PCR procedure was conducted in a volume of 20 μl, containing 2 μl cDNA, 0.4 μl of each primer, and a 10 μl ChamQ Universal SYBR qPCR Master Mix. The reaction was conducted in the ABI StepOnePlus Real-Time PCR System with Tower. The gene expression level was analyzed with 2^–ΔΔCT^ method. *Actin1* was used as an internal control to normalize the expression levels of different samples. All assays were performed in two independent experiments with three repetitions.

## Results

### Phenotypic evaluation

Significant and continuous variations of adaptive traits were exhibited in the diverse panel. The BLUE values of FLL, FLW, UIL, and PM (0 means prostrate, one means semi-prostrate, two means erect) were 25.0 cm (19.4–29.0 cm), 14.7 mm (10.8–19.1 mm), 22.3 cm (14.1–33.3 cm), and 2.70 (1.3–3.0). The standard deviation and coefficient of variation of FLL, FLW, UIL, and PM were 7.25 cm (0.29), 2.94 mm (0.20), 5.58 cm (0.25), and 0.59 (0.22). The UIL was negatively correlated with FLW (−0.557, *p* < 0.001). FLL with FLW, PM and UIL, FLW with PM, and UIL with PM show no significant correlation (Supplementary Table S4). ANOVA showed highly significant effects (*p* < 0.01) of genotypes, environments, and genotype × environment interactions on all traits (Table 1). The *h* ^2^ estimates by a one-stage approach for FLL, FLW, UIL, and PM were 0.78, 0.50, 0.93, and 0.79, whereas the SNP-based heritabilities for FLL, FLW, UIL, and PM were 0.77, 0.50, 0.91, and 0.78, respectively, indicating that morphological traits were determined by genetic factors and affected by environment.

**TABLE 1:** ANOVA analysis for the morphological traits in 251 spring wheat accessions from the Northeast of China.

Source of variation	*SS*				
	df	FLL	FLW	UIL	PM
Genotypes	250	7370.3**	6788.0**	65468.23**	303.5**
Environments	3	2274.3**	6726.8**	1187.8**	19.8**
Replicates (nested in environments)	8	144.2**	5717.06**	1133.7**	5.9**
Genotypes*Environments	750	5584.15**	8761.1**	17678.2**	193.4**
Error	1806				

*and ** indicate significance at 0.05 and 0.01 levels.

### Genotyping, population structure, and linkage disequilibrium decay analysis

Totally, 52,503 polymorphic SNPs after filtering (MAF <0.05, missing rate >0.1) were conducted for further GWAS (Li et al., 2021). Among the SNP markers, 18323, 18691, and 15489 were from A, B, and D genomes, respectively, with an average marker density of 0.273 Mb per marker. All the 251 spring wheat accessions could be divided into three subgroups, subgroups I, II, and III. Of these, subgroup I contained 126 varieties mainly from Heilongjiang ranging from 1950s to 1980s; subgroup II had 75 varieties mainly from Heilongjiang ranging from 1990s to 2010s; whereas subgroup III comprised 50 varieties mainly from Jilin and foreign counties, including United States, Canada, and Japan. Furthermore, the average LD for the whole genome was 8 Mb according to the LOESS curve (Li et al., 2021).

### Genome-wide association study

**FIGURE 1:**
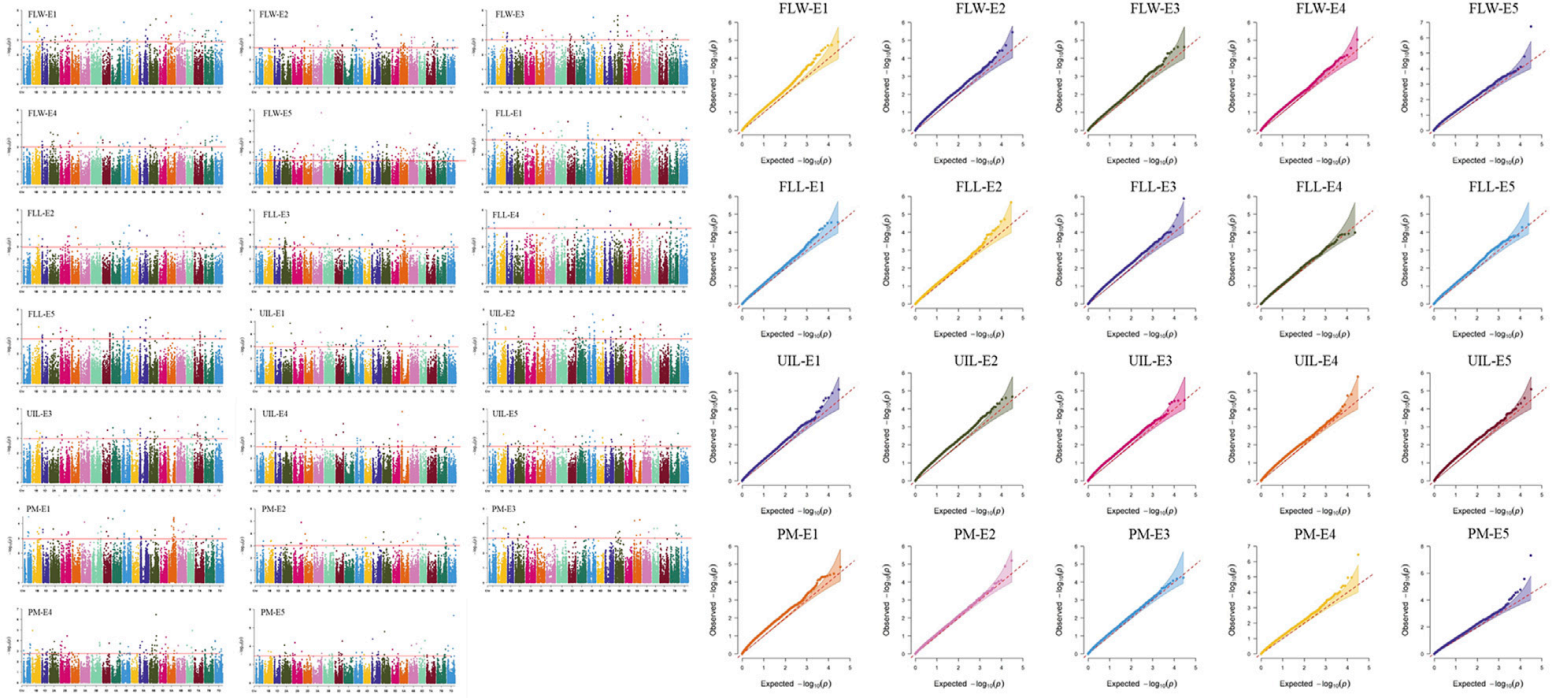
Manhattan and Q–Q plot for morphological traits in 251 spring wheat accessions from the Northeast of China analyzed by the mixed linear model (MLM) in Tassel v5.0. FLL, Flag leaf length; FLW, Flag leaf width; UIL, Uppermost internode length; PM, Plant morphology. The 1, 2, 3, 4, and 5 indicated the Harbin 2018, Harbin 2019, Keshan 2018, Keshan 2019, and the best linear unbiased estimation (BLUE), respectively.

Thirty loci were detected associated with morphological traits (Table 2; Supplementary Table S5; Figure 1; Supplementary Figure S1). Among these, nine loci for FLL were identified on chromosomes 1, 2, 2A, 2B, 3B, 3D, 5D, 6A, and 7D, and each explained 5.7–9.5% of the phenotypic variances, respectively; seven for FLW were identified on chromosomes 1A (2), 5A, 5A, 5B, 5D, and 7A, and explained 5.1–17.9% of the phenotypic variances, respectively; seven for FLL were identified on chromosomes 2B, 5A, 6A, 6D, 7A, 7B, and 7D and explained 4.8–8.8% of the phenotypic variances, respectively; seven for PM were identified on chromosomes 2B, 2B, 3D, 4A, 5A, 5B, and 6A, and each explained 5.3–12.5% of the phenotypic variances, respectively.

**TABLE 2:** Loci for morphological traits in 251 spring wheat accessions from the Northeast of China by association analysis.

Trait	Chr	Start (Mb)	End (Mb)	R^2^		*p*-value		Environment	Favorable allele	References
				Min	Max	Min	Max			
FLL	1A	572.9	577.7	5.9%	9.0%	2.6E-05	8.6E-04	E1, E3, E4, BLUE	C	Li et al. (2021)
FLL	2A	66.3	68.2	6.0%	9.5%	1.7E-05	8.7E-04	E4, BLUE	G	
FLL	2A	554.6	560.2	5.8%	7.3%	2.2E-04	9.3E-04	E4, BLUE	C	Li et al. (2021)
FLL	2B	652.7	656.0	6.1%	7.9%	1.4E-04	9.2E-04	E1, E2, E4, BLUE	C	Liu et al. (2018)
FLL	3B	121.4	129.6	5.8%	9.3%	1.8E-05	9.8E-04	E1, E2, E4, BLUE	G	Wu et al. (2016)
FLL	3D	82.8	94.5	5.9%	7.8%	1.2E-04	7.7E-04	E1, E2, E4, BLUE	G	
FLL	5D	551.2	556.2	5.9%	8.3%	5.5E-05	9.2E-04	E2, E4, BLUE	G	
FLL	6A	5.1	17.9	5.8%	8.3%	5.4E-05	9.2E-04	E1, E2, E4, BLUE	T	
FLL	7D	5.4	10.5	5.7%	7.0%	2.3E-04	9.8E-04	E1, E4, BLUE	A	Wu et al. (2016)
FLW	1A	296.9	297.7	5.8%	6.6%	3.3E-04	8.4E-04	E1, E3, BLUE	C	
FLW	1A	556.0	587.4	5.3%	8.1%	1.8E-05	7.0E-04	E1, E2, BLUE	A	Li et al. (2021)
FLW	5A	290.1	299.5	6.0%	7.7%	1.5E-04	8.9E-04	E1, E3, E4, BLUE	A	
FLW	5A	595.4	597.7	6.2%	7.7%	1.1E-04	5.3E-04	E2	A	Wu et al. (2016)
FLW	5B	615.7	617.9	5.1%	10.7%	9.0E-05	9.4E-04	E1, E3, E1, BLUE	A	Li et al. (2021)
FLW	5D	562.0	562.7	5.7%	9.3%	1.8E-05	9.2E-04	E1, E3	A	
FLW	7A	18.8	25.9	5.8%	17.9%	1.4E-09	9.5E-04	E2, E1, E3	G	
UIL	2B	409.9	439.5	5.1%	8.2%	1.8E-05	9.1E-04	E1, E2, E3, E4, BLUE	G	
UIL	5A	383.4	385.0	5.6%	8.3%	4.6E-05	9.3E-04	E2	G	Li et al. (2020)
UIL	6A	606.6	611.7	5.2%	5.9%	9.3E-04	9.5E-04	E1, E2, E3, E4, BLUE	G	
UIL	6D	388.2	407.5	4.8%	6.8%	2.5E-04	9.0E-04	E2, E4, BLUE	G	Li et al. (2020)
UIL	7A	496.3	512.9	5.0%	6.8%	2.8E-04	8.1E-04	E1, E2, E3, E4, BLUE	C	
UIL	7B	701.1	701.1	6.6%	8.8%	6.5E-06	1.3E-04	E1, E3, E4, BLUE	A	Li et al. (2020)
UIL	7D	8.3	8.3	4.9%	6.0%	3.1E-04	9.6E-04	E1, E3, E4, BLUE	A	
PM	2B	91	92.6	6.1%	7.7%	1.0E-04	7.8 E-04	E2	A	
PM	2B	663.1	664.2	5.9%	6.6%	3.9E-04	9.2E-04	E2, E4	G	
PM	3D	602.8	606.7	5.9%	8.4%	5.1E-05	8.3E-04	E1, E3, E4	G	
PM	4A	41.4	46.2	5.3%	12.5%	2.7E-06	3.7E-04	E2, E4, BLUE	C	Liu et al. (2016)
PM	5A	688.9	688.9	7.0%	9.9%	1.5E-06	4.4E-05	E1, E4, BLUE	C	
PM	5B	696.7	697	6.6%	8.9%	2.9E-05	5.0E-04	E1, E2, E3, BLUE	C	
PM	6A	23.8	24.4	6.9%	10.1%	7.5E-06	2.6E-04	E2, BLUE	C	Li et al. (2020)

FLL, flag leaf length; FLW, flag leaf width; UIL, uppermost internode length; PM, plant morphology.

The E1, E2, E3, E4, and E5 indicated the Haerbin 2018, Haerbin 2019, Keshan 2018, Keshan 2019, and the best linear unbiased estimation (BLUE), respectively.

Of these, the locus on chromosome 1A (556.0–577.7 Mb) is an identical locus, which showed effects on FLW and FLL; a locus on chromosome 2B (652.7–664.2 Mb) controlled both the FLL and ST; a locus on chromosome 5D (551.2–562.7 Mb) is the same loci for FLL and FLW. Besides, FLL and UIL have the same locus on chromosome 7D (5.4–8.3 Mb).

### Candidate genes

Totally, seven candidate genes for morphological traits were identified (Table 3). Two candidate genes encoded for the E3 ubiquitin-protein ligase-like protein (*TraesCS1A01G164400* and *TraesCS2B01G466600*) were identified in the LD decay of the loci on 1A (296.9–297.7 Mb) and 2B (652.7–656.0 Mb) chromosomes. Another gene encoding a cytokinin riboside 5′-monophosphate phosphoribohydrolase (*TraesCS1A01G362500*) was identified in the LD decay of the loci on chromosome 1A (556.0–587.4 Mb). For the loci on chromosome 5A (383.4–385.0 Mb and 595.4–597.7 Mb), candidate genes *TraesCS5A01G182500* and *TraesCS5A01G406800* were identified, which encode the F-box family protein. Besides, the genes *TraesCS2B01G123800* and *TraesCS4A01G055000* encoding the C2H2 zinc finger and leucine-rich repeat receptor protein kinase were identified as the candidate gene for the loci 2B (91.0–92.6 Mb) and 4A (41.4–46.2 Mb). The qRT-PCR of seven candidate genes showed that *TraesCS2B01G466600* and *TraesCS5A01G406800* showed no significant differences between the extreme accessions; *TraesCS1A01G164400*, *TraesCS4A01G055000,* and *TraesCS1A01G362500* showed 1.6–3.2-fold higher expressions among the extreme accessions, whereas *TraesCS5A01G182500* and *TraesCS2B01G123800* showed more than 1.9–7.6-fold lower expressions among extreme accessions (Supplementary Figure S1).

**TABLE 3:** The details for the candidate genes of morphological traits.

Candidate gene	Chromosome	Start	End	Annotation
*TraesCS1A01G164400*	1A	296212311	296215426	E3 ubiquitin-protein ligase
*TraesCS1A01G362500*	1A	543067408	543069241	Cytokinin riboside 5′-monophosphate phosphoribohydrolase
*TraesCS2B01G123800*	2B	91304955	91305926	Zinc finger, C2H2
*TraesCS2B01G466600*	2B	662376739	662379204	E3 ubiquitin-protein ligase
*TraesCS4A01G055000*	4A	46141864	46145610	Leucine-rich repeat receptor protein kinase
*TraesCS5A01G182500*	5A	382291561	382292892	F-box protein-like protein
*TraesCS5A01G406800*	5A	597793675	597796893	F-box family protein

## Discussion

All the 251 spring accessions could be divided into three subgroups (Li et al., 2021), and the characterization of the subgroups was largely consistent with geographic origins, released years, and pedigrees. Most of the cultivars from Heilongjiang ranging from 1950s to 1980s belonged to subgroup-1; the accessions from Heilongjiang ranging from 1990s to 2010s belonged to subgroup-2, whereas subgroup-3 mainly including the accessions from the Jilin and foreign counties (United States, Canada). Population analysis (PCA, NJ-tree, and Kinship) indicated that a significant population structure existed in the 251 spring wheat accessions. The lack of appropriate correction for population structure can lead to spurious MTAs (Zhu et al., 2008). Thus, to eliminate spurious MTAs, an MLM model with subpopulation data (Q) (fixed-effect factors) and kinship matrix (random-effect factor) was conducted. LD decay was influenced by population structure, allele frequency, recombination rate, and selection progresses and significantly affected the precision of association mapping. The LD decay for the whole genome was about 8 Mb, consistent with previous studies (Liu et al., 2017). The number of markers is enough for the subsequent association analysis.

### Comparison with the QTL or gene in previous studies

The genes or loci associated with morphological traits were extensively reported previously (Wu et al., 2016; Li et al., 2018; Li et al., 2021). In this study, the association of morphological traits was performed and 30 significant loci were detected.

Several studies for flag leaf-related traits in common wheat have reported. We have identified nine loci for FLL in eight different chromosomes. Li et al. (2020) have identified eight loci for FLL on chromosomes 1A, 2A (2), 2B, 3A, 5A, 6B, and 6D, and each explained 6.9–19.6% of the phenotypic variances. Of these, the loci identified in the 1A and 2A were overlapped with the regions 1A (572.9–577.7 Mb) and 2A (554.6–560.2 Mb) identified in this study. Wu et al. (2016) mapped two FLL QTL on chromosomes 3B and 7D, which is overlapped with the FLL locus (3B: 121.4–129.6 Mb and 7D: 5.4–10.5 Mb) in the present study. Another FLL QTL was previously identified on chromosome 2B linked with the SSR marker *barc318* (Liu et al., 2018); it is coincided with the present FLL locus (2B: 652.7–656.0 Mb) based on the consensus linkage map (Maccaferri et al., 2015). These loci on chromosomes 2A (66.3–68.2 Mb), 3D (82.8–94.5 Mb), 5D (551.2–556.2 Mb), and 6A (5.1–17.9 Mb) were potential novel loci. Li et al. (2020) have identified five loci for FLW on chromosomes 1A, 3B, 5B (2), and 6B, accounting for 6.9–11.4% of the phenotypic variances. The locus on chromosome 5B (*AX_109519234*) was coincided with the locus (5B: 615.7–617.9 Mb) detected in this study. Besides, the locus on chromosome 1A (296.9–297.7 Mb) was also overlapped with the locus on 1A (*AX_111540798*) identified by Li et al. (2018). Wu et al. (2016) reported a locus for both FLW and flag leaf angle at the position (*IWB4576*) on chromosome 5A, showing a pleiotropic effect, which coincided with the loci identified in this study (5A: 290.1–299.5 Mb). In conclusion, the loci identified in 1A (556.0–587.4 Mb), 5A (595.4–597.7 Mb), 5D (562.0–562.7 Mb), and 7A (18.8–25.9 Mb) were potentially new.

UIL is important for the construction of plant architecture and influences the yield. Several studies have focused on the genetic basis of UIL. Wu et al. (2016) have identified seven loci for UIL. Li et al. (2020) have identified 12 loci for UIL on chromosomes 1A (2), 1B (2), 3A, 5A, 6B (3), 6D (2), and 7B, with a single locus explaining 6.7–16.4% of the phenotypic variances. Of these, the loci 1A (*AX_109449226*), 6D (*AX_109331000*), and 7B (*AX_186165710*) coincided with 5A (383.4–385.0 Mb), 6D (388.2–407.5 Mb), and 7B (701.1 Mb) identified in this study. Besides, the UIL locus (6D: 388.2–407.5 Mb) is about one LD from the QTL associated with both PH and the third internode length (Cui et al., 2011); they are likely to be the same. Except for the three loci talked above, the remaining four loci (2B: 409.9–439.5 Mb; 6A: 606.6–611.7 Mb; 7A: 496.3–512.9 Mb, and 7D: 8.3 Mb) are all likely to be novel.

Until today, a few studies have been conducted on plant morphology for common wheat. Thus, it is difficult to compare with the present results. Li et al. (2002) centered to the Gli-A2 gliadin locus and associated with a QTL affecting prostrate growth trait on chromosome 6A and nearly with the locus identified in this study in 6A (23.8–24.4 Mb) according to Maccaferri et al., 2015. Liu et al. (2016) have identified a region associated with plant morphology on the 4A chromosome, which maybe coincided with the locus identified in our study (4A: 41.4–46.2 Mb). Although some studies have been focused on the plant habit growth in bread wheat, no marker information was available in order to confirm our results. Thus, we think the loci 2B (91–92.6 Mb, 663.1–664.2 Mb), 3D (602.8–606.7 Mb), 5A (688.9 Mb), and 5B (696.7–697 Mb) were potential be novel.

Among the 30 loci for morphological traits, 13 loci talked above (1A: 572.9–577.7 Mb, 2A: 554.6–560.2 Mb, 2B: 652.7–656.0 Mb, 3B: 121.4–129.6 Mb, 7D: 5.4–10.5 Mb, 1A: 296.9–297.7 Mb, 5A: 290.1–299.5 Mb, 5B: 615.7–617.9 Mb, 5A: 383.4–385.0 Mb, 6D: 388.2–407.5 Mb, 7B: 701.1–701.1 Mb, 4A: 41.4–46.2 Mb, and 6A: 23.8–24.4 Mb) should be the same as the QTL reported in previous studies, whereas the remaining 17 are likely to be new. The stable loci validated by our studies and previous studies indicated that they are widespread and maybe stable in various varieties.

### Candidate gene analysis

To identify candidate genes for morphological traits, the flanking sequences of SNP markers (in the LD decay interval and corresponding non-synonymous SNPs in the coding region of the genes) significantly associated with morphological traits were used as queries to BLAST against the NCBI. Totally, seven candidate genes were identified. For loci 1A (296.9–297.7 Mb) and 2B (652.7–656.0 Mb), the candidate genes for the E3 ubiquitin-protein ligase-like protein (*TraesCS1A01G164400* and *TraesCS2B01G466600*) were identified in the LD decay distance. E3 ubiquitin-protein ligase-like protein plays a crucial role in plant growth and development (Karki et al., 2021; Zhang et al., 2022). For loci 4A (41.4–46.2 Mb), candidate gene *TraesCS4A01G055000* encoded leucine-rich repeat receptor-like protein kinase family protein, which may trigger multiple physiological pathways (Coleman et al., 2021). For the loci on chromosome 5A (383.4–385.0 Mb and 595.4–597.7 Mb), candidate genes (*TraesCS5A01G182500* and *TraesCS5A01G406800*) for F-box proteins were identified. F-box proteins play crucial roles in cell-cycle progression, transcriptional regulation, flower formation, signal transduction, and many other cellular processes (El-Sharkawy et al., 2021; Guérin et al., 2021). Of these, a cytokinin riboside 5′-monophosphate phosphoribohydrolase (*TraesCS1A01G362500*) was identified in the LD decay of the loci on chromosome 1A (556.0–587.4 Mb). The cytokinin is a positive regulator of shoot growth (Chen et al., 2021) and response to biotic and abiotic stressors (Ellis et al., 2005; Chen et al., 2022). Besides, the gene *TraesCS2B01G123800* was identified as the candidate gene for the loci 2B (91.0–92.6 Mb) and encodes the C2H2 zinc finger, which is important for determining the prostrate/erect plant morphology (Li et al., 2021). The expressions of seven candidate genes showed that *TraesCS2B01G466600* and *TraesCS5A01G406800* showed no significant differences between the extreme accessions; *TraesCS1A01G164400*, *TraesCS4A01G055000,* and *TraesCS1A01G362500* showed 1.6–3.2-fold higher expressions among the extreme accessions; whereas *TraesCS5A01G182500* and *TraesCS2B01G123800* showed more than 1.9- to 7.6-fold lower expressions among extreme accessions. Thus, *TraesCS1A01G164400*, *TraesCS4A01G055000*, *TraesCS1A01G362500*, *TraesCS5A01G182500,* and *TraesCS2B01G123800* are the candidate genes in our study.

### Potential implications in wheat breeding

Although conventional breeding has led to improved morphological traits on wheat, selection is time-consuming and not very efficient (He et al., 2010; Rasheed et al., 2016). The SNPs associated with morphological traits identified in this study should facilitate the progresses of MAS and pyramiding favorable alleles will improve morphological traits (Li et al., 2018). Accessions with superior morphological traits and high numbers of favorable alleles (such as Hechun 5, Jichun 158, LongFu 18-171, LongFu 17-5277-12-24 and Longchun 1 with longer FLL; Xinshuguang 4, Dongnong 156597, Kechun 151350, Kenda 163672 and Gang 09-558 with wider FLW; Longmai 17, Kehua, Xiaomaixiaobing 4, Xiaomaixiaobing seven and Yongjie with longer UIL; Kehan 1, Shen 68-71, Mailiduo, Jichun101 and Jichun1 with erect, whereas Beimai6, Lianfeng, Kechun 140865, Beimai one and LongFumai8 with prostrate) could be used as parental lines for the improvement of morphological traits in wheat breeding. The loci with pleiotropic and consistent effects across each environment in this study should be amenable to MAS.

## Conclusion

We have identified 30 loci for morphological traits in spring wheat accessions by GWAS. Of these, 17 loci were likely to be new. Besides, five candidate genes were identified for morphological traits. The associated markers and varieties with favorable alleles could be used to accelerate the progresses of wheat MAS breeding.

## Data Availability

The original contributions presented in the study are included in the article/Supplementary Material; further inquiries can be directed to the corresponding author.

## Supplementary material

SUPPLEMENTARY FIGURE S1 The qRT-PCR results of the seven candidate genes for the extreme accessions of the 25 spring wheat accessions from the Northeast of China. The extreme accessions of the 25 spring wheat accessions from the Northeast of China were according to Supplementary Table S2.

## Abbreviations

BLUE, Best linear unbiased estimation; FLL, Flag leaf length; FLW, Flag leaf width; PM, Plant morphology; GWAS, Genome-wide association study; hb2, Broad-sense heritability; KASP, Kompetitive allele-specific PCR; LD, Linkage disequilibrium; MAS, Marker-assisted selection; QTL, Quantitative trait loci; R2, Phenotypic variance explained; RIL, Recombinant inbred line; SNP, Single nucleotide polymorphism; UIL, Uppermost internode length.
