# Supplementary figures and images for: Genetic analysis of morphological traits in spring wheat from the Northeast of China by a genome-wide association study
Source: Front Genet. 2022 Aug 18;13:934757. doi: 10.3389/fgene.2022.934757 (PMC9434797; doi:10.3389/fgene.2022.934757)

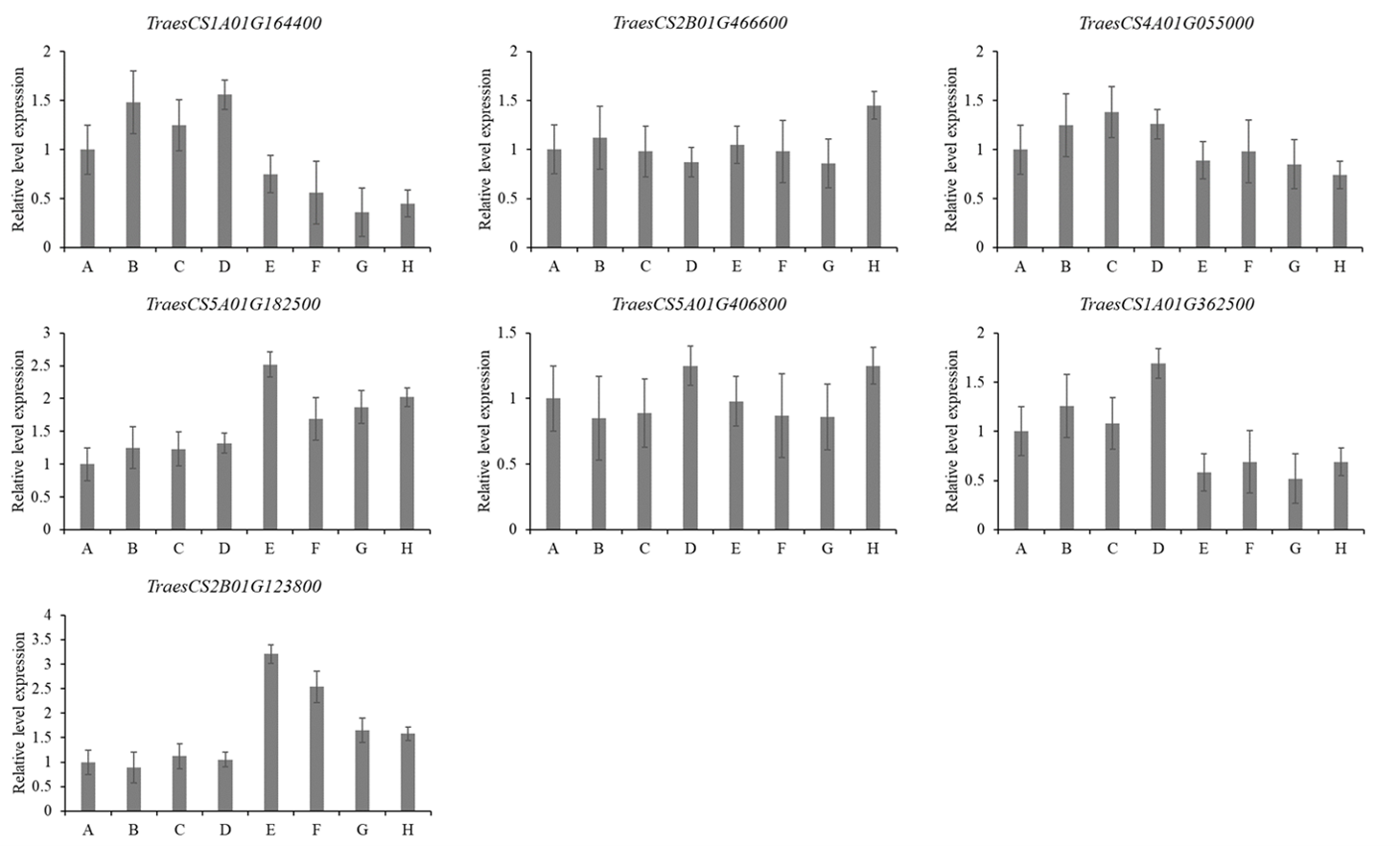

Supplement: Supplementary file 1 [file Image1.tif]
